# IDGenerator: unique identifier generator for epidemiologic or clinical studies

**DOI:** 10.1186/s12874-016-0222-3

**Published:** 2016-09-15

**Authors:** Matthias Olden, Rolf Holle, Iris M. Heid, Klaus Stark

**Affiliations:** 1 Department of Genetic Epidemiology, Institute of Epidemiology and Preventive Medicine, University of Regensburg, Regensburg, Germany; 2 Institute of Health Economics and Health Care Management, Helmholtz Zentrum Munich, Neuherberg, Germany

**Keywords:** Identifier, ID, ID generator, ID creator, Unique, Barcode, Check digit, Epidemiologic study, Clinical study

## Abstract

**Background:**

Creating study identifiers and assigning them to study participants is an important feature in epidemiologic studies, ensuring the consistency and privacy of the study data. The numbering system for identifiers needs to be random within certain number constraints, to carry extensions coding for organizational information, or to contain multiple layers of numbers per participant to diversify data access. Available software can generate globally-unique identifiers, but identifier-creating tools meeting the special needs of epidemiological studies are lacking. We have thus set out to develop a software program to generate IDs for epidemiological or clinical studies.

**Results:**

Our software IDGenerator creates unique identifiers that not only carry a random identifier for a study participant, but also support the creation of structured IDs, where organizational information is coded into the ID directly. This may include study center (for multicenter-studies), study track (for studies with diversified study programs), or study visit (baseline, follow-up, regularly repeated visits). Our software can be used to add a check digit to the ID to minimize data entry errors. It facilitates the generation of IDs in batches and the creation of layered IDs (personal data ID, study data ID, temporary ID, external data ID) to ensure a high standard of data privacy. The software is supported by a user-friendly graphic interface that enables the generation of IDs in both standard text and barcode 128B format.

**Conclusion:**

Our software IDGenerator can create identifiers meeting the specific needs for epidemiologic or clinical studies to facilitate study organization and data privacy. IDGenerator is freeware under the GNU General Public License version 3; a Windows port and the source code can be downloaded at the Open Science Framework website: https://osf.io/urs2g/.

## Background

In epidemiological studies, identifiers (IDs) are unique tokens used to mark study participants and their study data [1]. The most straight forward approach is to utilize serial or random numbers or characters as IDs. However, epidemiological studies often require more sophisticated solutions.

First, study recruitment may be conducted sequentially for numerous reasons requiring the generation of IDs in batches: a ***consecutive batch of IDs*** needs to be controlled for being distinct from existing IDs. Second, organizational aspects often call for a more structured approach: ***structured IDs*** carry not only a random identifier, but also organizational information. Examples for such information are a study center in the case of multi-center studies or information as to what study program a participant pertains (called in the following “study track”). In some instances, it may be of interest to code the visit number, if the participant visits the study center multiple times (for example to distinguish between baseline, follow-up, or regularly repeated visits or for applications like biobanking, where bio-samples from the same user may be acquired at different time points). Finally, a check code might be of interest to detect data entry errors.

Third, the scientific best practice requires separate storage of personal data from study data. The rationale is that study data can be sensitive (e.g. including severe disease diagnoses, life style information) and should be kept separate from personally identifiable information (name, birth date, address). For some tasks (report study results to participants, re-contacting of participants), linking both sides is mandatory. As employed by many studies including the German National cohort [2] and KORA [3], one approach is to have multiple IDs to diversify the data access (***layered IDs***): one ID for personal data (ID-P), another for study data (ID-S) and different IDs for data to be transferred to external partners (ID-E). A possible model may involve granting very restricted access to ID-P for recruiting and study personnel, access to ID-S for study analysts to facilitate quality control, and different ID-Es to external partners for data analysis to avoid re-identification and merging of study data between different external partners. The mapping of the different IDs is usually only temporarily required, e.g. for producing results reports that are to be sent to the participant or for re-contacting in the case of longitudinal studies. When generating these multi-layered IDs, a concept for ID linkage is mandatory.

There are several software packages like EpiInfo [4], OpenEpi [5], EpiData[6], Askimed [7] or OpenClinica [8] that provide basic frameworks to design case-report forms for entering study data, but none includes the generation of structured and layered IDs. Other software tools e.g. the Online GUID Generator [9] create globally unique identifiers (GUIDs) [10], which do not guarantee uniqueness but are most likely unique per design: by selecting randomly from a large enough pool (128 bit), the probability of identical GUIDs is very small (close to zero). There are also tools that compute check digits, like GS1 Check Digit [11] or Bulk Check Digit Calculator [12], these however are oriented towards commercial applications like Global Trade Item Numbers instead of epidemiologic studies.

We developed a software program that guarantees unique IDs, supports the generation of structured IDs to facilitate study organization, provides layered IDs to enhance data protection, and can extend existing IDs with new non-overlapping batches. While IDGenerator was originally developed for the needs of the AugUR study [13], it allows for different parametrization and therefore can be applied to epidemiological studies with different requirements.

## Implementation

### Use case in the AugUR study

The German AugUR study (Age-related diseases: understanding genetic and non-genetic influences - a study at the University of Regensburg) is a prospective study targeted towards the elderly mobile population in Bavaria. The aim of the study is to recruit 3,000 random participants aged 70 or older and patients selected from the University Hospital Regensburg, phenotype these in respect to eye and cardiovascular diseases and conduct follow-up analyses after 3 years. Each participant was to be assigned a unique ID containing a number coding the study (to distinguish from other studies in our institute), a number coding the study track (local registry of residence based, clinic-based, or volunteers), a unique participant number (5-digits), a number or a character coding the study visit and a check digit. We created a total of 14,000 IDs to be used during the recruitment stage (20–25 % response rate yielding 3,000 participants). As study data is stored separately from personally identifiable information, two distinct IDs (ID-S for study data and ID-P for personal data) were needed. Also, the clinical results for the participants and the cover letter with name and address were printed from two systems and manually mapped over a temporary ID (ID-T).

### Comparison against semi-manual techniques

As random IDs can also be generated with standard office programs such as Microsoft Excel, we first attempted to use standard tools to perform the steps required to produce 14,000 random IDs for the AugUR study. We created 100,000 random non-unique numbers using the RANDBETWEEN function, filtered about 30,000 unique results and selected 14,000 numbers out of these. We then concatenated the coding digit for our study number, study tracks, study visits and computed a simple check digit using the MOD and MID functions. We could not compute complex check digits or barcode formats without Excel programming. While this may be a solution for very small studies (e.g. up to 1,000 participants), it has several drawbacks: it is limited by the Excel capabilities per worksheet (e.g. only 1,048,576 random non-unique numbers can be created) [14], it cannot easily extend the existing IDs or add new tracks, and it is error-prone due to the complexity of the steps required to be performed by a human operator. This motivated us to implement a simple automated software solution for solving these issues.

### Overall software architecture

The key task of IDGenerator software is the generation of IDs for epidemiological studies providing the necessary flexibility and modern features for data protection and data entry error detection: create unique random IDs, support various options to define a wide range of patterns for structured IDs, provide layered IDs, or generate new batches of IDs, that are distinct from existing IDs.

A graphical user interface supports the software utilization in a user-friendly manner. In four steps, the user can (i) define the ID structure, (ii) specify parameter settings, (iii) select the specific task, (iv) and run the program. The output lists the IDs in two formats, one for entry into an electronic record file system and another for generating bar codes.

### Ensuring uniqueness of generated identifiers

The key feature of the software is to ensure the uniqueness of generated identifiers. The software uses a pseudo-random number generator class that can yield a sequence of numbers complying with statistical requirements for randomness (lacking any recognizable pattern). The random function is initialized with a seed representing the number of milliseconds since the computer has started. IDGenerator supports the definition of the random number length, constraints to the interval, from which the numbers or characters are to be chosen, and the selection of new batches of IDs controlling for them being distinct from previously selected IDs.

Speed is a critical issue for larger sample sizes (more than five digits), as any newly generated random ID needs to be examined to ensure it differs from every previously created ID. Considering the often applied mode of ID generation for all persons contacted (to facilitate non-response analyses) rather than only generating IDs for all persons actually agreeing to participate, it is necessary to generate two to ten times as many IDs compared to the number of actual study participants (considering a response fraction between 50 and 10 %). A study with 10,000 participants would therefore need to compute 100,000 IDs taking into account a response rate of 10 %. Thus, the number of generated IDs becomes high rather quickly.

A tightly chosen interval for the sample size also affects the speed of ID generation algorithm. When the requested sample size is close or equal to the maximum number of available samples, the probability of randomly drawing duplicates increases significantly and more drawings are necessary until a new unique number is randomly found. For each newly drawn number, the list of previously generated numbers needs to be searched and compared with the new number to avoid duplicates. This process tends to become rather slow as the list grows due to the default comparison method involved. Thus, two variables are checked for identity (e.g. a = 123, b = 123, memory address 0000007B) using reference equality, which means that the program engine will scan the entire computer memory to see if the two variables refer to the same object in the memory.

An approach to accelerate the search is to use a string representation of numbers and perform a byte-by-byte comparison (e.g. for a = ***1***23, b = ***2***23, only the first bytes “1” vs. “2” are checked) to asses for actual object equality, checking whether the string representations of numbers equal each other. This method is faster, as it compares only parts of the string representation and returns that two numbers are different upon encountering the first different digit in the numbers.

### Concept of layered IDs

Good Clinical Practice (GCP) guidelines recommend separating personal data information from study data information to ensure protection for human subjects data [15]. This is often facilitated by generating layered IDs [16] in form of a personal ID (ID-P) used as unique identifying key to personally identifiable information and a study data ID (ID-S) used as unique identifying key to scientific data.

There are several approaches to link ID-P and ID-S. Our approach is to generate a temporary ID (ID-T) and create two mapping files: one containing the (ID-P, ID-T) key pair, the other containing the (ID-S, ID-T) key pair. The two mapping files are ideally stored in two separate systems - with the (ID-P, ID-T) mapping file being the one that should be stored in a particularly secure system with restricted access and without internet connectivity. During the study conduct, which can be several years or even decades for longitudinal studies, the ID-T is utilized for linking the information (pseudo-anonymized for data analysis). At the end of the study, the ID-T can be deleted from all files, which facilities the anonymization of the study data meeting the highest level of data protection.

### Concept of structured IDs

Another key feature of IDs in epidemiological studies is the fact that one might prefer to code some organizational information into the ID. Our software tackles this issue by enabling different patterns of blocks that form the ID, with the mandatory block being the random number. Optional blocks are a code for study center (for multi-center studies), for study track (e.g. cases or controls), or for the visit number in the study center.

If the study program differs between subjects, different study tracks may be also encoded into the ID, e.g. depending on how the participant was recruited (from local registries of residence, general practitioners, or clinics) or depending on participant characteristics (sex, age-group). However, the coding of participant characteristics into the ID should be only used with care to avoid re-identification [1].

The visit number may be also encoded into the ID in order to distinguish between multiple records belonging to the same participant (e.g. when labeling bio-materials). Yet, it should be noted that coding the visit number into the ID is less widely applied and, instead, identical IDs across visits (with an additional variable like examination date coding for the number of visit) are often used [17].

### Control for ID entry error

Besides organizational information, another block can be added that provides a check digit to detect data entry errors in the case that the ID is entered manually [18]. Depending on the specific algorithm, check digits can detect single digit errors (e.g. one digit typed wrong), format errors (one digit wrongly inserted or omitted) or transpositions (two digits switched). The challenge in implementing any of these algorithms is not only to add the check digit to the ID, but also to implement consistency checks into other programs that test the check digit correctness when the ID is entered.

We implemented the most widely applied algorithms for check digits:

1. With the *parity check method* [18], the check digits is computed as modulo 10 of the sum all digits of the ID. For letter digits, the American Standard Code for Information Interchange (ASCII) code associated to the letter (e.g. 65 for “A”) is used. This method is the easiest to double check or implement, but does not detect transpositions (two consecutive digits switched).
2. The *weighted parity check* [18] computes the module 10 of the sum of all digits, where each digit is multiplied with a number specifying its position. This method can detect adjacent transpositions, but not non-adjacent transpositions.
3. With the algorithms Gumm_1986 [19] and Damm_2004 [20], non-adjacent transpositions can be detected. However, these approaches are the most complex to re-implement.

### Technical implementation

The technical implementation of the software is driven by the organizational structure of the study center. In this case, the software requirements specifications were: usable by study personnel without programming skills, independent of previous installation or software dependencies, simple to understand Windows interface, and low hard- and software demands for running on offline personal computers due to data protection reasons.

IDGenerator was developed under Visual Studio.Net 2012, as this allows a standard Windows graphic user interface (GUI), try-catch error handling and an easy installation without package dependencies. The minimum screen resolution is 1024×768 pixels. The output is in form of ASCII text files and configuration files are stored in eXtensible Markup Language (XML) text format. The software is compatible with both 32 bit and 64 bit Intel processor architectures.

**Fig. 1:**
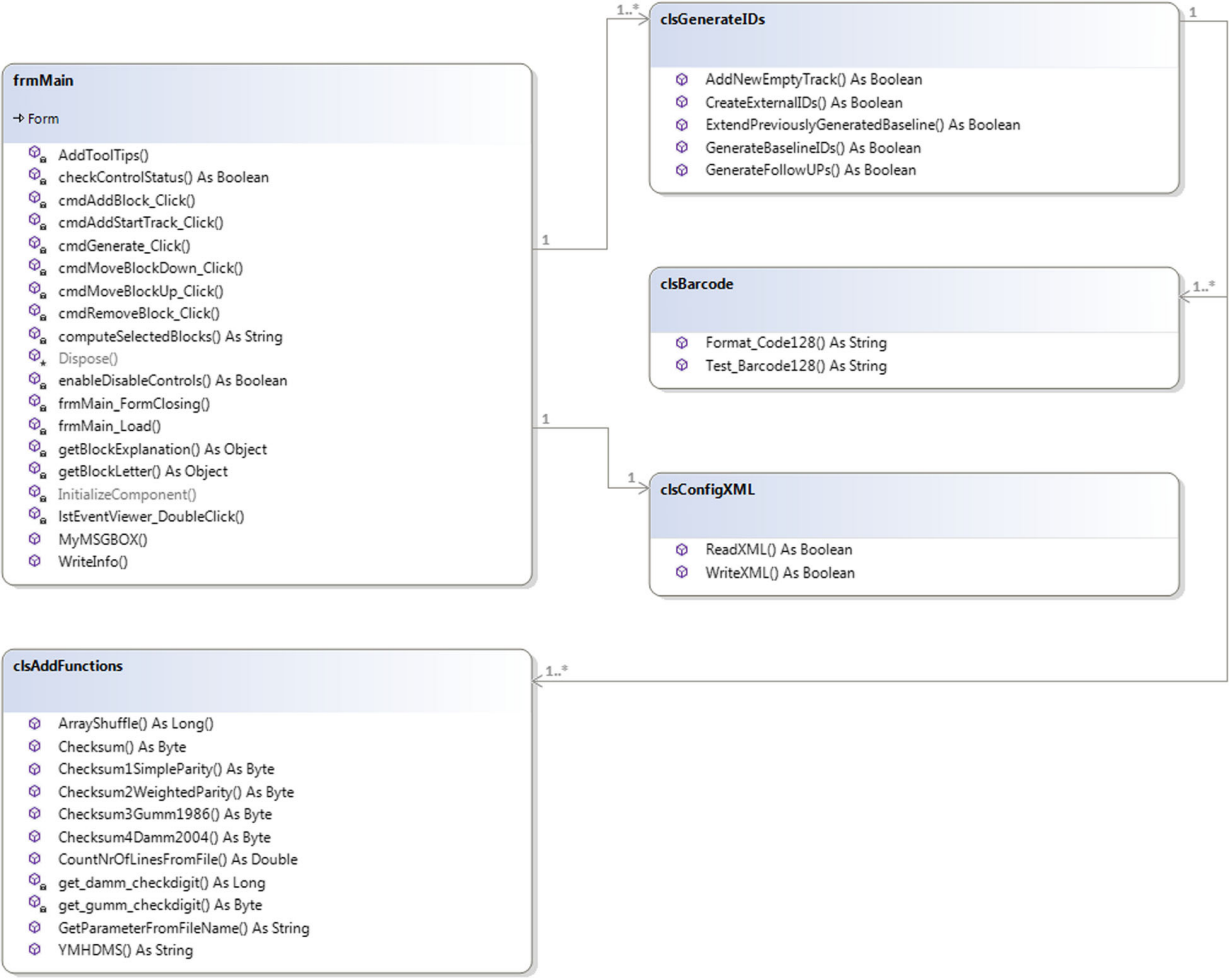
UML class diagram of the idGenerator software. The IDGenerator code contains the following classes: *frmMain* (overall functionality and GUI commands, shared variables), *clsGenerateIDs* (creates baseline IDs, extends previously created IDs, creates follow-up IDs or generates external IDs), *clsBarcode* (creates barcode 128B readable data), *clsAddFunctions* (help functions), *clsConfigXML* (functions for the configuration file)

The IDGenerator code is object-oriented and contains the following classes (Fig. 1):

- *frmMain* – implements the overall functionality and GUI commands; stores shared variables;
- *clsGenerateIDs* – implements methods for creating new (baseline) IDs, extends previously created baseline IDs, creates follow-up IDs based on baseline data or generates external IDs for data sharing;
- *clsBarcode* – implements functions for creating barcode 128B readable data;
- *clsAddFunctions* – implements help functions, such as check digits, file naming using date-time functions, data reads and writes, and performs plausibility checks;
- *clsConfigXML* – implements read and write functions for the configuration file.

The process of ID generation consists of 3 steps: in a first step (“CHECK”), plausibility checks test the quality of each user input value. All selected blocks must not be empty or contain special characters (like empty spaces), track names must be unique, valid sample sizes must be entered for all selected tracks and the total number of requested combination must be lower than the number of possible combinations for the given number size.

In the second step (“GENERATE”), the program allocated 3 arrays (for ID-P, ID-S and ID-T) of the total sample size requested for all tracks and starts generating random numbers using the Random() class constructor as implemented in.Net to initialize the random number generator with a time-dependent seed value. To accelerate the process of checking newly drawn random IDs, the program uses the *Array.Contains()*.NET function to check if a drawn number has already been selected, which is considerably faster than sequentially searching the available number sets for yet un-selected numbers. This function uses the enumeration rule StringComparison.Ordinal, which compares strings based on binary sorting rules.

Finally, in the third step (“SAVE”), the additional information (study center, study track, study visit) is added to the random number and a check digit is computed according to the user input from step 1. The data is immediately stored in text format and discarded from memory.

## Results

The functionalities of IDGenerator encompass the full workflow of designing, creating, extending and managing IDs for epidemiological studies and are described below.

### Layered IDs

**Fig. 2:**
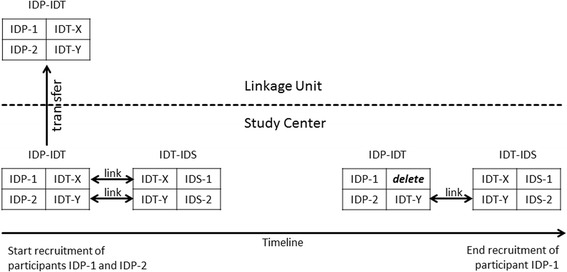
Concept of layered IDs. The study center creates two key files (ID-P, ID-T) and (ID-S, ID-T) before recruitment and transfers a copy of the (ID-P, ID-T) file to a trusted linkage unit. Later in the recruitment phase, the study center may delete the ID-T from the (ID-P, ID-T) key file for already recruited participants and detach the link to the study data. The link may be reconstructed using the original key file from the linkage unit. The study may also choose to exchange the (ID-S, ID-T) list Instead of the (ID-P, ID-T), if the ID-P list requires additional protection and cannot be exchanged

IDGenerator implements the concept of layered IDs by separating the personal ID-P from the study ID-S into different files and linking these over a common temporary ID-T. The personal file contains the key pairs (ID-P, ID-T) and the study file contains the key pairs (ID-S, ID-T), where the values for ID-T are the same in both files (Fig. 2). The study center creates both key pairs files before the recruiting begins and may choose to transfer a copy of the (ID-P, ID-T) key file to a linkage unit for storage. Later in the study recruitment phase, the study center may delete the ID-T from the (ID-P, ID-T) key file for already recruited participants or non-responders and thus detaching the link to the study data identified by the (ID-S, ID-T) key file. In case of recontacting, the linkage unit can provide the deleted ID-T information based on a list of ID-Ps. The study may also choose to exchange the (ID-S, ID-T) list instead of the (ID-P, ID-T), if the ID-P list requires additional protection and cannot be exchanged.

### Blocks for structured IDs

The structure of the IDs is composed of following parts (blocks): [C] study center, [T] study track, [N] a unique random number, [V] study visit and [X] check digit. With the exception of the unique random number, all other blocks are optional. Upon selection, the blocks move from the list of available blocks to the list of selected blocks, where they can be arbitrarily sorted. The selection [C] allows the generation of IDs for one study center with the center name being part of each ID. The selection [T] allows for generating IDs for one or multiple study tracks (e.g. cases or controls, men or women) with the study track names being part of the ID. The selection [V] allows for generating IDs with the same unique [N] number and with a new visit number, in order to distinguish records for the same participant at different time points. The selection [X] adds one check digit generated from all other digits based on a specific algorithm to check for data entry errors.

### Parameter settings

All blocks have features to configure, some being specific to an optional block:

(i) In any case, the *study name* is required, which is used for naming the directory to which the identifiers are stored on disk (general feature).
(ii) In any case (general feature), a *sample size (n)* is required, which defines the number of IDs to be generated. If multiple tracks are specified, sample sizes for multiple tracks must be provided separately with semicolon).
(iii) In any case, the *length of the random numbers* (*k*) must be specified.
(iv) If the block [C] is selected, the *name of the center* is to be specified and will be used in the ID code (e.g. if the chosen feature for study center is “9” and the [C] is the first block, all IDs will start with “9”). IDs will be generated for this one center. If IDs are to be generated for a second center, the procedure has to be repeated.
(v) If the block [T] is selected, the *name of the track(s)* are to be specified and will be used in the ID code (e.g. if the chosen setting for tracks are “1; 2” and [T] is the second block, then ID batches will contain “91” and “92”).
(vi) If the block [V] is selected, the *code of the visit* is to be specified.
(vii) If [X] is selected, the specific *check digit algorithm* is to be specified (parity check, weighted parity check, Gumm_1986 method [19] and Damm_2004 method [20]). The check digits are natural numbers.

### Random numbers in the identifiers

The random numbers [N] in the ID are natural numbers within [1 × 10^k^; 4 × 10^k^[for ID-P, within [4 × 10^k^; 7 × 10^k^[for ID-S, and within [7 × 10^k^; 10 ×10^k^[for ID-T (fixed intervals). For example, if a 5-digit random number is requested (*k* = 5), a maximum of 30,000 IDs can be generated, with the random number for ID-P from [10,000; 40,000[, for ID-S from [40,000; 70,000[, and for ID-T from [70,000; 100,000[. To achieve this, IDGenerator defines a new instance of the Random class, with a time-dependent default seed value taken from the Environment.TickCount() property, representing the number of milliseconds passed since the computer was started. The random numbers are then created using the Random.Next(*lower_bound*, *upper_bound*) function, which yields natural numbers within the boundaries of the range specified by *lower_bound* and *upper_bound*.

### The main tasks of the software

#### Create IDs

IDGenerator creates *n* random numbers of the length *k* by drawing a random number for each of the ID-P, ID-S and ID-T from the respective interval and selecting the number, only if it is distinct from any previously selected numbers (within one study). The requested codes for study center, study track and check digits are added in the order and with the parameters previously specified. For each of the ID-P, ID-T and ID-S, the same study center name and track name is used, but different random IDs are assigned. The visit is always “0” for ID-P and takes on natural numbers for ID-T and ID-S. The pairs (ID-P, ID-T) and (ID-S, ID-T) are stored in standard and in barcode 128B format in a directory named after the study name. The pair (ID-P, ID-T) is stored as created; for the pairs (ID-S, ID-T), the order is randomized to prevent a re-association simply by the order in the files. The files are stored as:

- [STUDYNAME]_IDP_IDT_T = [TRACK]_N = [SAMPLESIZE]_Baseline and
- [STUDYNAME]_IDS_IDT_T = [TRACK]_N = [SAMPLESIZE] _Baseline in ASCII text format.

#### Add new IDs

When the originally requested IDs are all used and new ones are required, a new batch of IDs can be generated, again controlling the new IDs to be distinct from previously selected ones. IDGenerator creates any new ID (if maximum number was not reached), checks for uniqueness from all previously generated IDs (for this one study), and produces the two ID lists (ID-P, ID-T) and (ID-T, ID-S) as described previously. The existing ID files are renamed by renaming their extension from “.txt” to “.old”, and the new ID batch is stored as:

- [STUDYNAME]_IDP_IDT_T = [TRACK]_N = [NEW_SAMPLESIZE]_Baseline and [STUDYNAME]_IDS_IDT_T = [TRACK]_N = [NEW_SAMPLESIZE]_Baseline.

#### Adding new visit

When the block [V] is selected, a new batch of IDs can be generated for a new visit. The visit name is, again, specified by the user (see specification of parameter settings). IDGenerator checks whether the requested visit name has been already used. The new IDs are the same as the previous IDs except for the part of the ID that codes the visit, which now carries the new visit (and, eventually, a new check digit). No new ID-P and ID-T is generated as these remain the same for all visits. Instead, a file with key pairs of ID-S (at first visit) and ID-S at the new visit is created. For example, if the baseline (visit = “***1***”) pairs of (ID-S, ID-T) for three participants are (451***1***, 8021), (651***1***, 9071) and (578***1***, 7281), and the new visit is called “***A***”, the new file will contain (451***1***, 451***A***), (651***1***, 651***A***) and (578***1***, 578***A***). This file is stored as is stored as:

- [STUDYNAME]_IDS_IDS***A***_T = [TRACK]_N = [SAMPLESIZE]_V = ***A***.

#### Add new track

When the block [T] is selected, new tracks may be added to the existing ones. IDGenerator checks whether the requested track name has already been used. It generates new empty pairs of (ID-P, ID-T) and (ID-S, ID-T) and saves these in a file:

- [STUDYNAME]_IDS_IDT _T = [NEW_TRACK]_N = 0_Baseline.

This option is only implemented out of technical reasons and should be combined with the option **Add new IDs**.

#### Generate external IDs

External IDs are created from the key pair (ID-S, ID-T) in form of (ID-S, ID-E) key files, where ID-S is common for both files. They consist of three parts: a project ID, a random number of length *k + 1* and a check digit (used only if ID-S employs also check digits). To create external IDs, IDGenerator first loads the key pair files (ID-S, ID-T), (ignoring the ID-T part), then generates the external ID-E from a larger pool of numbers as ID-S (e.g. if ID-S has *k* = 5 digits, the ID-E will have 6 digits for *k*), attaches the project ID to the random number and applies the same check digit method as used for ID-S. For example, if the key file (ID-S, ID-T) is: (4511, 8021), (6511, 9071) and (5781, 7281), with the random number [N] of length *k* = 3 digits followed by visit [V] = 1 and without check digit, the file for an external project “EXT” will contain the key pair (ID-S, ID-E) file as: (4511, EXT8825), (5781, EXT8042) and (6511, EXT9114). The numbers of ID-E contain the project name “EXT” followed by 4-digit random numbers and without check digits. These key pairs are stored in the file:

- [STUDYNAME]_IDS_IDE_T = [TRACK]_N = [SAMPLESIZE]_Prj = EXT.

### The graphical user interface

**Fig. 3:**
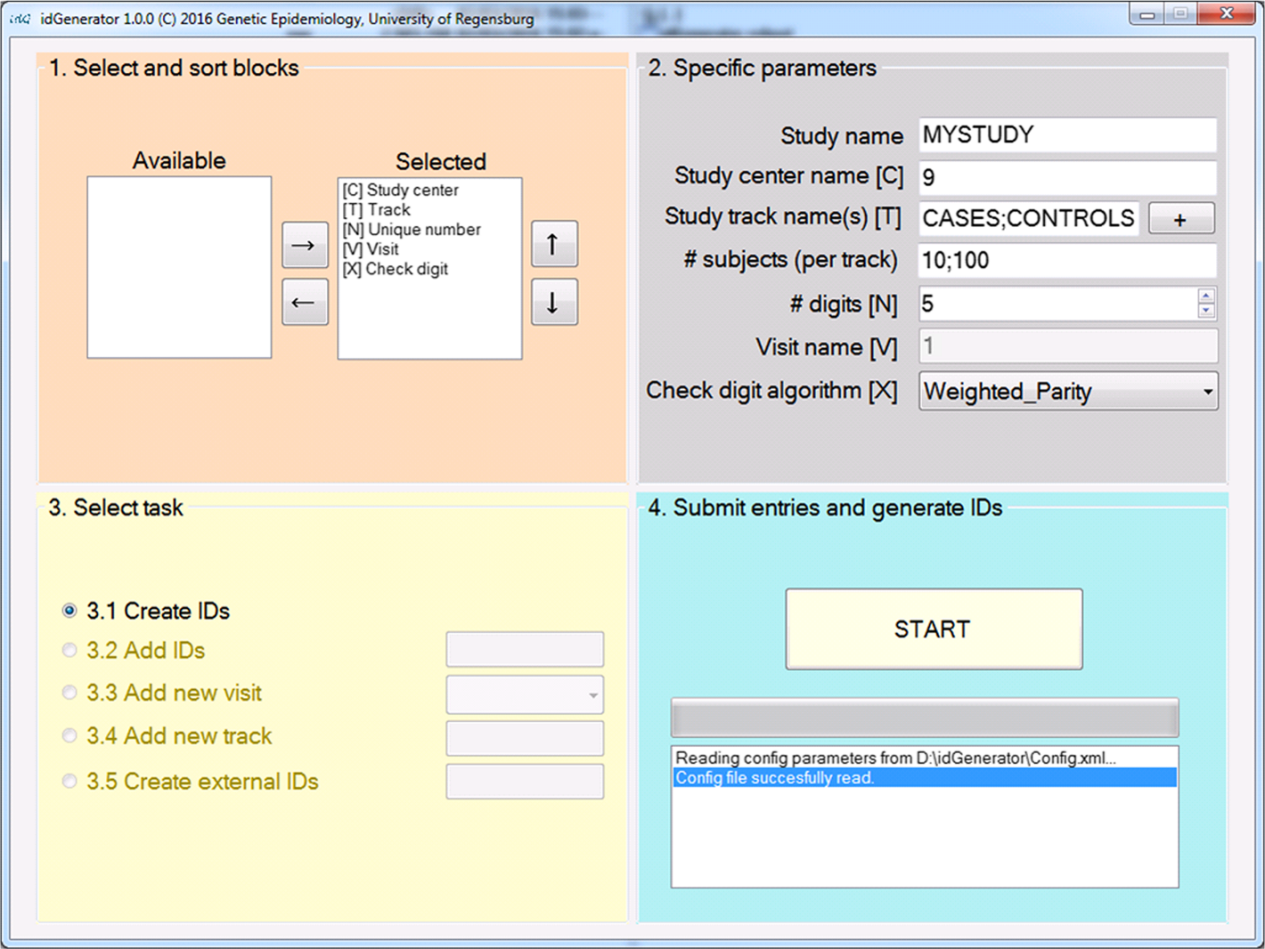
IDGenerator graphical user interface. The interface is organized in four compartments in-line with the four workflow steps: (1) Select and sort blocks: The respective blocks can be selected from an available list and then sorted. (2) Specify parameters: Provide a study name (for the directory naming, number or characters, no spaced allowed), a study center name (number or character, no spaced allowed), track name(s) (number or characters, no space allowed, multiple tracks separated by semicolons), the number of subjects for which IDs are requested (per track, in the case of multiple tracks separated by semicolons), random number length (values between 2 and 9), visit name (numbers between 1–9 or characters, not allowed are “i", “e”, “o” or special characters, case sensitive, default visit is 1), and the algorithm to create check digits. (3) Specify the task. When the program is used for the first time in a study, the first task is necessarily task 1 “Create IDs”. (4) Submit entries and generate IDs. After pressing the “START” button, the software will start computing the IDs (duration depending on chosen settings). A progress bar will show the percentage of generated IDs

The IDGenerator workflow involves four steps, which are reflected in a user-friendly interface (Fig. 3):

1. Select and sort blocks: The respective blocks can be selected and sorted.
2. Specify parameters: Provide a study name (for the directory naming, number or characters, no spaced allowed), a study center name (number or character, no spaced allowed), track name(s) (number or characters, no space allowed, multiple tracks separated by semicolons), the number of subjects for which IDs are requested (per track, in case of multiple tracks separated by semicolons), random number length (values between 2 and 9), visit name (numbers between 1 and 9 or characters, not allowed are “i", “e”, “o” or special characters, case sensitive, default visit is “1”), and the algorithm to create check digits.
3. Specify the task. When the program is used for the first time in a study, the first task is necessarily task 1 “Create IDs”.
4. Submit entries and generate IDs.

All entries into the GUI are stored in an.xml file and recalled upon restart.

## Discussion

The IDGenerator software allows a fast generation of study identifiers for small to medium epidemiologic studies, with all processing steps done in the computer random-access memory. The numbers generated are guaranteed to be unique, their check digits enable the detection of user input errors, and the barcode format representation endows IDs to be read by barcode scanners. Also, although originally developed for epidemiological studies, IDGenerator may be also used in the setting of clinical studies.

Some limitations warrant mentioning. The maximum number of IDs is limited by the maximum size of arrays. In.Net and other programming languages (like Java), array lengths are limited to the highest integer 32 bit value, the largest value that can be represented in 32-bit two's complement. This enables theoretically 2,147,483,647 (2^31^–1) unique combinations, out of which, for *k* = 9 digits, IDGenerator can create a maximum of 300,000,000 unique ID key pairs, corresponding to all numbers from [100,000,000; 400,000,000[for ID-P, all numbers from [400,000,000; 700,000,000[for ID-S, and for all numbers from [700,000,000; 1,000,000,000[for ID-T. As all ID-P, ID-S and ID-T are distinct from each other, this results in 300,000,000 × 3 = 900,000,000 unique IDs. A *k* = 10 digits would result in a total number of 9,000,000,000 IDs, which is higher than the maximum of 2,147,483,647 combinations that may be stored into arrays. For studies requiring more than 300,000,000 unique key pairs, multiple instances of the software using different study centers (e.g. study center “1”, study center “2”, a.s.o.) may be used to produce larger unique numbers.

Another problem encountered when dealing with large unique randomly-generated numbers is speed. In case the requested number of IDs is close to this maximum number of possible IDs or the number of requested combinations is large (k > 6 or more than 1,000,000 combinations requested), IDGenerator may take a long time to randomly pick these numbers. This is due to the fact that, for each new random number generated, this must be compared to the entire array of previously generated numbers to ensure uniqueness. This process takes seconds for k < 6 (tens of thousands of IDs), hours for k = 6 (hundreds of thousands of IDs) or days for k > 6 (millions of IDs) on a personal computer with an Intel® Core™ i7-3770 @ 3.4 GHz with 16 GB of RAM memory and running Windows 7 Professional Service Pack 2. These times vary with the memory space and processor speed available and are necessary to ensure a qualitative ID which is guaranteed to be unique. Multiple study centers, study tracks or complex check algorithms do not affect the time performance of the software.

One option to speed up the ID generation would be serial number drawing. However, a single key set of (ID-P, ID-S) and (ID-S, ID-T) is enough to derive subsequent IDs. E.g. the key pairs (241***0***, 907***1***) and (651***1***, 907***1***) with *k* = 3 digits and visit = ***1*** (***0*** for ID-P) may be used to determine the next key sets: (242***0***, 908***1***) and (652***1***, 908***1***). This option is therefore not implemented in IDGenerator, as it would conflict with the concept of layered ID separation.

Another option of accelerating the creation process for large numbers is by using permutation algorithms like Fisher-Yates-Shuffle [21], which first generate a sequential array of numbers and then shuffle every element to a random position.

A third option for fast ID generation is to create just a part of the total number of IDs and extend the ID pool with new IDs when needed. In case of multiple study centers, multiple instances of the software with distinct study center [C] could generate in parallel parts of the overall IDs. Our software is designed to facilitate such approaches.

There is also potential for further advancement. For example, the software may be converted from.Net to another programming language such as Java or Python, if the study intends to use it on other operating systems such as UNIX.

Our software is designed to accommodate enough IDs for currently running or prospective epidemiologic or clinical studies. In case future studies would need to use more IDs than arrays can store, the software may be adapted to handle large numbers as text and store them into clusters of text files instead of arrays. This method would have the advantage that it may be parallelized, but would need a computer cluster or computer cloud to run instead of a standard desktop computer. The generation of random numbers in the cloud will require separation into chunk intervals of numbers to avoid duplicates. Furthermore, studies may need approval from ethics committees to generate sensitive information such as IDs in the cloud.

Also, there may be potential scenarios when studies would need to include other options such as user-specified intervals for all layers of IDs, other barcode types (e.g. Code 39) or even other types of IDs (e.g. own ID-B for biobank).

In its current form, IDGenerator addresses towards small to medium epidemiologic or clinical studies in need of a simple yet secure concept and tool for ID creation management. The software may be used by study personnel without programming training and on a standard Windows computer.

## Conclusions

IDGenerator provides an automated tool to generate IDs with multiple features, particularly for modern epidemiological or clinical studies. The software enables the generation of structured IDs to facilitate study organization, layered IDs to enhance data protection, and check digits to detect entry errors. It runs without installation on Windows systems, requires no programming skills to use, and provides IDs as standard text and 128B barcode.

## Abbreviations

ASCII: American standard code for information interchange
C: Study center
GUID: Globally unique identifier
ID: Identifier
ID-B: Biobank identifier
ID-E: Identifier for data to be transferred to external partners
ID-P: Personal data identifier
ID-S: Study data identifier
ID-T: Temporary identifier
N: Unique random number
T: Study track
V: Study visit
X: Check digit
XML: Extensible markup language
